# Gait variability during abrupt slow and fast speed transitions in older adults with mild cognitive impairment

**DOI:** 10.1371/journal.pone.0276658

**Published:** 2022-10-21

**Authors:** Sirinun Boripuntakul, Teerawat Kamnardsiri, Stephen Ronald Lord, Surinthorn Maiarin, Puangsoi Worakul, Somporn Sungkarat

**Affiliations:** 1 Faculty of Associated Medical Sciences, Department of Physical Therapy, Chiang Mai University, Chiang Mai, Thailand; 2 Research Group of Modern Management and Information Technology, College of Arts, Media and Technology, Chiang Mai University, Chiang Mai, Thailand; 3 Department of Digital Game, College of Arts, Media and Technology, Chiang Mai University, Chiang Mai, Thailand; 4 Neuroscience Research Australia, School of Public Health and Community Medicine, University of New South Wales, Sydney, New South Wales, Australia; 5 Clinical Psychology Program, Faculty of Education, Prince of Songkla University, Pattani Campus, Pattani, Thailand; University of Rochester, UNITED STATES

## Abstract

Gait speed modulation, including abruptly decreasing or increasing gait speed, is a challenging task and prerequisite for safe mobility in the community. Older adults with Mild Cognitive Impairment (MCI) exhibit gait deficits under challenging walking conditions which may increase their risk of falls. The purpose of this study was to investigate spatiotemporal variability during slow and fast speed transitions in older adults with and without MCI. Twenty-five older adults with MCI (mean age = 68.56 ± 3.79 years) and 25 cognitively intact controls (mean age = 68.72 ± 4.67 years) participated. Gait performance during gait speed transitions was measured in two walking conditions: 1) a slow to fast speed transition in response to a randomly presented cue, and 2) a fast to slow speed condition in response to a randomly presented cue. Means and variability of spatiotemporal parameters during the transitions were measured and mixed model repeated measures ANOVAs were used to assess interaction and main effects. The older adults with MCI exhibited greater variability of step length (MCI = 13.93 ± 5.38, Control = 11.12 ± 3.15, p = 0.03) and swing time (MCI = 13.35 ± 6.01, Control = 10.43 ± 2.87, p = 0.03) than the controls during the fast to slow speed transitions. No other between-group differences were evident for the gait parameters across the two walking conditions. The findings suggest that older adults with MCI have reduced ability to adapt their gait during transitions from fast to slow walking speeds. This impairment may indicate a decline in automated regular rhythmic gait control and explain in part why this group is at increased risk of falls. Slow speed transition task might be incorporated as a fall risk screening in older adults with MCI.

## Introduction

Mild Cognitive Impairment (MCI) is a condition in which an individual has cognitive deficits exceeding normal aging that are not sufficient to meet the diagnostic criteria for dementia [1]. A growing body of evidence suggests that in addition to cognitive dysfunction, older adults with MCI have gait deficits when compared to age-matched cognitively intact controls [2]. Moreover, older adults with MCI experience a larger decline in cognitive and gait functions over time than their cognitively-intact peers [3]. Cognitive and gait functions appear to be closely related, and both cognitive and gait impairments are well-documented risk factors for falls [4]. Since cognitive and gait impairments often coexist in older adults with MCI, the investigation of gait performance under challenging walking tasks relevant for daily life may assist in devising specific interventions for preventing falls in this group.

The ability to increase and decrease speed is important for safe mobility in the community. There are many situations for which individuals need to adjust their gait speed in response to environmental demands such as speeding up during crossing a street or slowing down to change direction or avoid an upcoming hazard [5]. Thus, examining the ability to make walking speed transitions may provide important information regarding gait adaptability in older adults. Previous studies have found that older adults are less able to increase their gait speed and stride length when required to walk at a fast speed compared to young adults, [6,7] and that older adults demonstrate impaired gait during gait termination [8,9].

As gait speed transitions, both to increase and decrease speed, require high levels of physical and cognitive control, it could be expected that older adults with MCI have difficulty in regulating gait during abrupt shifts to faster or slower speeds. Two studies have found older adults with MCI have impaired gait during gait initiation and termination compared to cognitively intact controls [10,11] and we have reported preliminary findings that older adults with MCI have greater step width and swing time variability than controls during slow to fast speed transitions [12]. No studies, however, have contrasted both fast and slow speed transitions between older adults with MCI and cognitively intact controls in a fully powered study design.

The aim of this case-control study was to compare gait characteristics during fast and slow speed transitions in response to randomly presented transition cues between older adults with MCI and cognitively intact controls. We hypothesized that older adults with MCI would have more variable gait (indicating reduced integrity of central neuromuscular system in maintaining a steady walking pattern) during both speed transition tasks compared to controls. This information may assist in the development of more comprehensive gait assessments and strategies for fall prevention in older adults with MCI.

## Material and methods

### Participants

The sample size was based on pilot study findings that yielded effect sizes of 0.43, 0.31, 0.20 for the coefficient of variation (CV) of swing time, step length, and step time respectively. With 80% power and a 5% type I error, a sample size of 50 participants (25 older adults with MCI and 25 cognitively intact controls) was required for the CV measure with the smallest effect size (step time).

Twenty-five participants with MCI aged 60 years or older and living independently in the Chiang Mai community were subsequently recruited via advertisements. Each participant was assessed by a psychologist to establish a diagnosis of MCI [13] based on established criteria: (a) presence of memory complaints from the patient and/or informant, (b) impaired cognitive performance in one or more cognitive domains (e.g. memory, attention, executive function), (c) preserved general functional independence, (d) absence of clinical dementia (determined by a Mental State Examination T10 (MSET10) score adjusted for educational level) [14]. In addition, to be eligible, all MCI participants needed to score ≤ 24 on the Montreal Cognitive Assessment (MoCA) [15].

Each participant with MCI was matched with a cognitively intact control based on age (plus or minus 1 years), gender, and body mass index (BMI) (plus or minus 1 units). Control participants were also living independently in the Chiang Mai community and recruited via advertisements. Common inclusion criteria for both groups were: ability to walk at least 10 meters without a walking aid and to follow test administration instructions. Common exclusion criteria were: presence of orthopaedic, neurological, cardiorespiratory or other unstable medical conditions that affected walking or safety during testing (e.g. Parkinson’s disease, stroke, peripheral neuropathy, uncontrolled hypertension, active asthma, acute lower back or lower limb pain), uncorrected visual and/or hearing deficits, depressive symptoms (determined by a Geriatric Depression Scale-15 score > 6) [16], or current use of sedative and antipsychotic drugs. The study protocol was approved by the Human Research Ethics Committee, Faculty of Associated Medical Sciences, Chiang Mai University (certificate of approval: AMSEC-61EX-087), and all participants gave written informed consent prior to enrolment in the study.

### Baseline assessments

Participants were interviewed about demographic characteristics and health conditions including age, educational level, co-morbidities, medical conditions, medication usage, and history of falls in the preceding 12 months. Fear of falling was assessed with the Falls Efficacy Scale-International (FES-I) for which higher scores indicate higher concern about falling [17]. Participant’s weight and height were measured to calculate BMI. Leg length of each participant was assessed from the anterior superior iliac spine (ASIS) to the tip of medial malleolus while standing using a flexible tape measure. To assess functional mobility, participants completed the Timed Up and Go test (TUG) [18]. For cognitive function, participants completed assessments within cognitive domains associated with gait deficits and falls including attention (assessed with the Digit Span Test and Trail Making Test Part A) and executive function (assessed with the Trail Making Test Part B-A) [19,20].

### The gait speed transition assessments

A three-dimensional (3D) motion analysis system (Motion Analysis^®^, Motion Analysis Corporation, Santa Rosa, California, USA) and EVaRT 5.0 software were used to capture gait characteristics of the participants during the gait speed transitions. The motion analysis system utilized ten infrared cameras (Eagle-4 camera system, Motion Analysis^®^) with a sampling rate of 120 Hz. The coordinate data were digitally filtered using a 4^th^ order low-pass Butterworth filter for 6 Hz cut-off frequency. Each spatiotemporal parameter was analysed using custom-built scripts in MATLAB (The Mathworks Inc of Natick, Massachusetts, USA).

**Fig 1:**
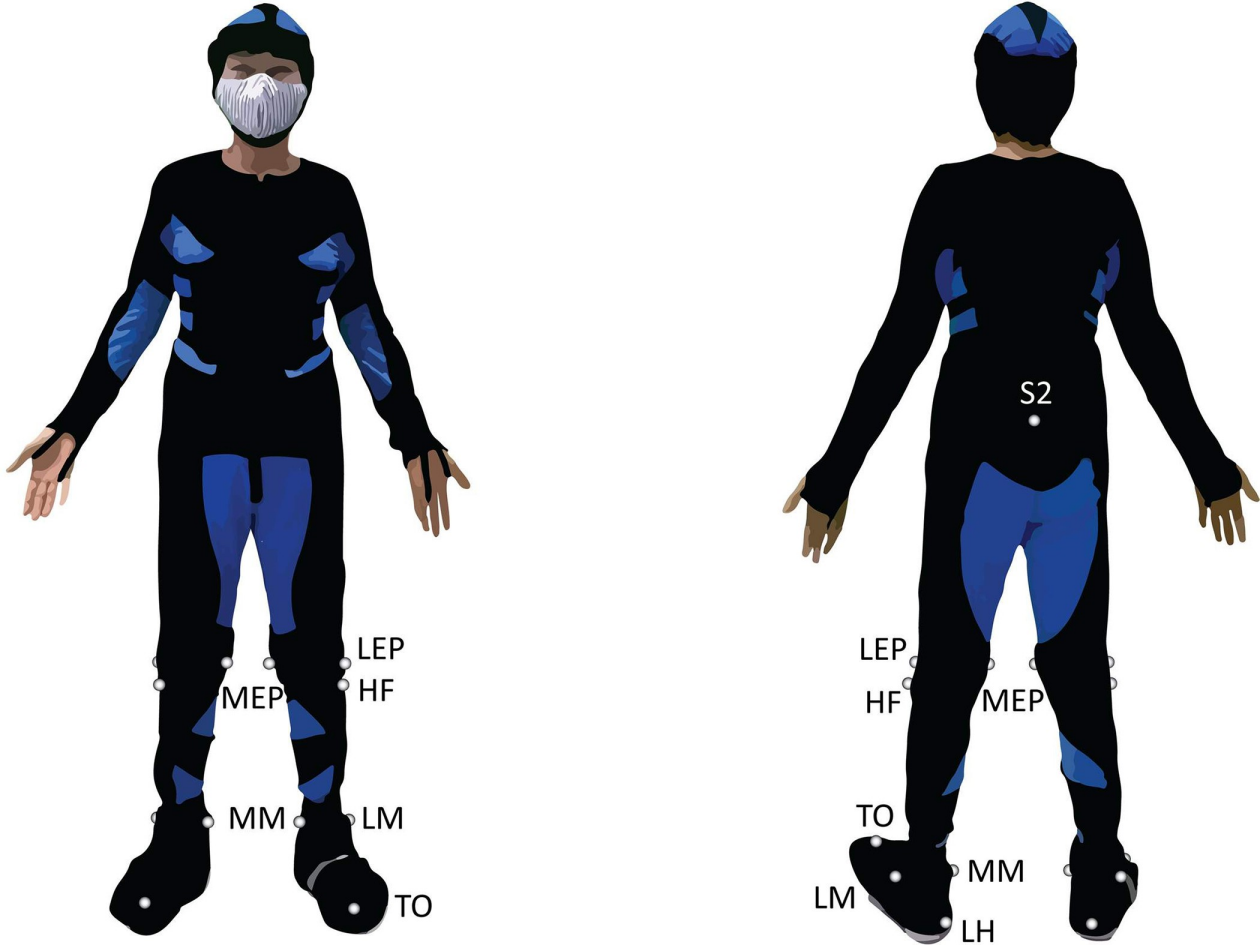
Placement of reflective markers on the lower extremities, S2 = second sacral vertebrae; MEP = medial epicondyle; LEP = lateral epicondyle; HF = head of fibula; MM = medial malleolus; LM = lateral malleolus; TO = head of second metatarsal; LH = calcaneus posterior inferior.

Fourteen reflective markers (2.0 cm diameter) were placed bilaterally onto the following anatomical landmarks of the lower extremities: medial and lateral epicondyles (MEP, LEP), head of fibula (HF), medial and lateral malleolus (MM, LM), head of second metatarsal (TO), and calcaneus posterior inferior (LH) [21] (Fig 1). Another marker was placed on the second sacral vertebrae (S2), which approximates the body’s center of mass, and used to track speed of body movement during walking.

Initially, participants performed two trials of self-selected slow, usual, and fast walks along a 10-meter walkway. Then in a fully randomised design, participants completed four slow to fast speed transitions (SF) and four fast to slow speed transitions (FS) within 24 catch walking trials where no transitions were elicited (i.e. 32 trials in total).

**Fig 2:**
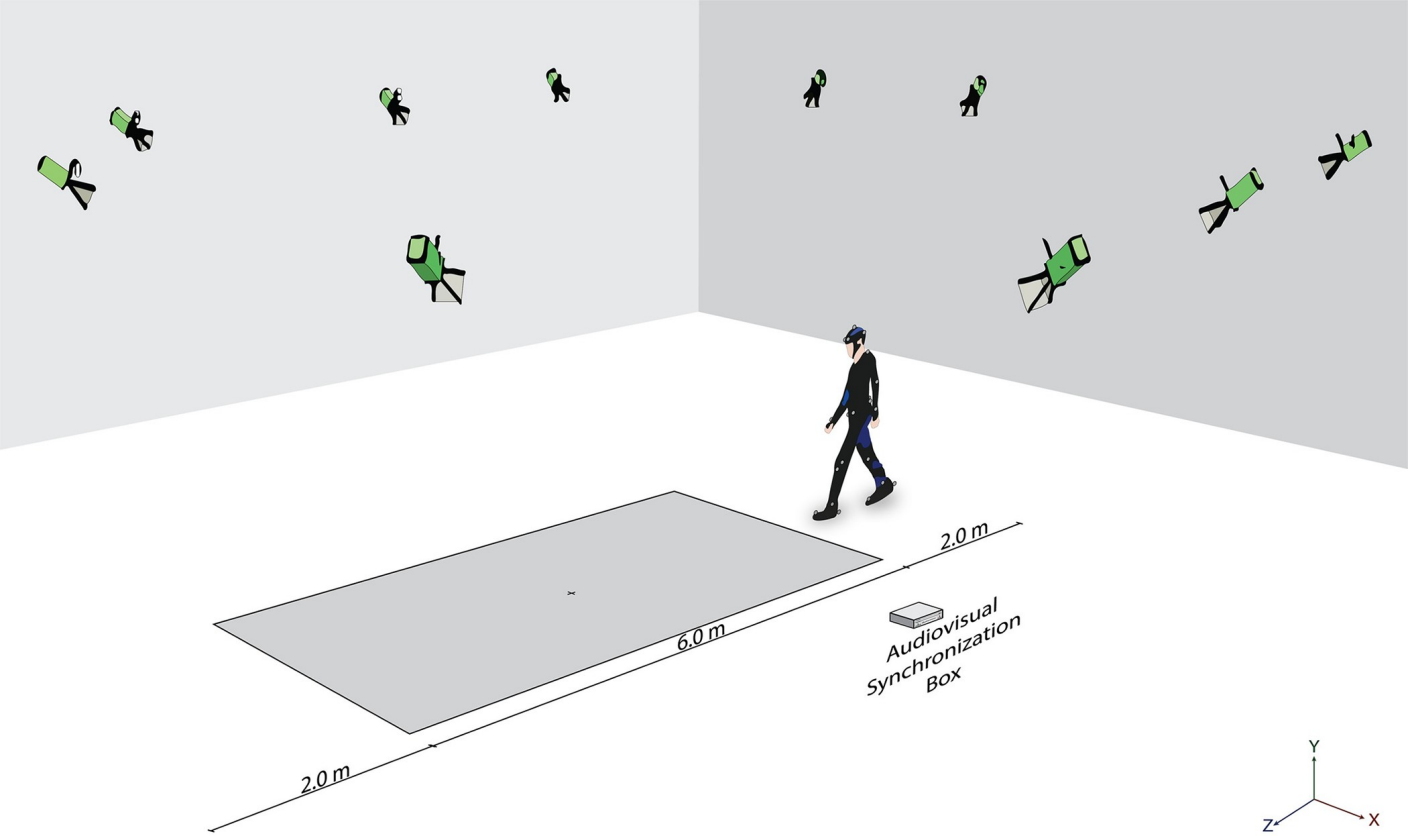
Experimental setup of gait speed transition assessment using 3D motion analysis system.

Two meters were provided prior to and following the capture zone to exclude the acceleration and deceleration walking phases; thus gait data were obtained from the middle 6-meters of the walkway (Fig 2). Participants were instructed to walk at an assigned speed (i.e. fast or slow speed) and increase or decrease their gait speed quickly in response to an auditory cue command (‘Fast’ or ‘Slow’) manually activated at a random time point using an audiovisual synchronization box (patent number: 16706) and then continue walking at the new speed until the end of the walkway. A demonstration was given to the participants before testing and standard instructions were given throughout the trials. A rest period was provided between each trial.

### Gait measure definition

**Fig 3:**
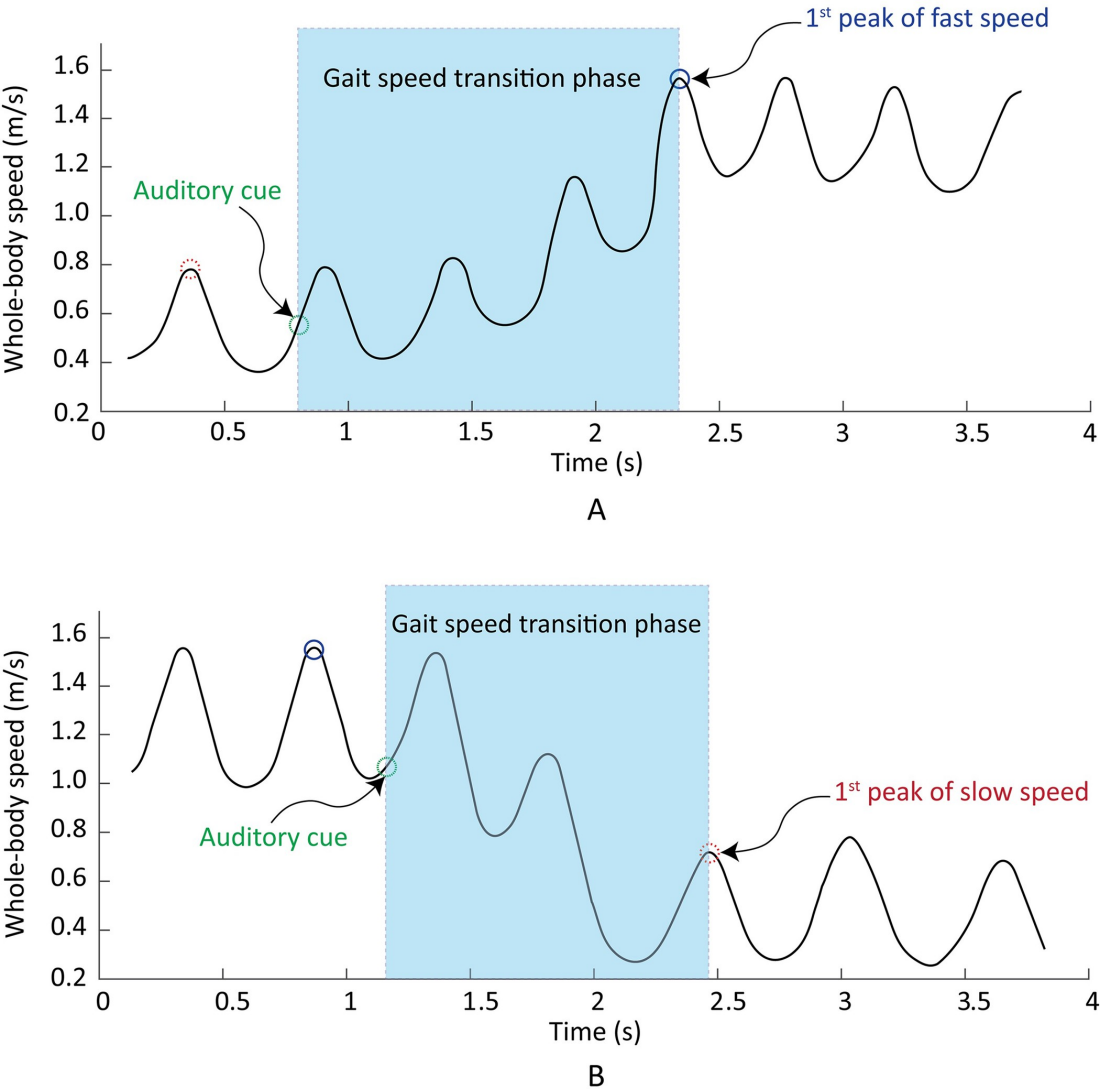
Gait speed transition phase: (A) SF condition (B) FS condition.

The gait speed transition period, tracked by the S2 marker, was defined as the interval between the presence of the auditory cue (‘Fast’ or ‘Slow’) to the point when a steady-state level of fast speed (SF condition) or slow speed (FS condition) was observed (Fig 3). Step length, step width, step time, and swing time means and standard deviations were computed using custom-built scripts in MATLAB. In prior studies, these gait parameters have been found to be indicators of rhythmic and dynamic gait control as well as risk of falls [22–24].

The mean and variability gait speed transition measures were quantified by averaging the data from the 4 walking trials within each walking condition. The coefficient of variation (CV) was used as the measure of gait variability with the equation CV = (standard deviation / mean) x 100. CV was calculated from all steps recorded during 4 walking trials (≈12–16 steps), an acceptable step number for test-retest reliability and concurrent validity [25].

Step length (cm) was defined as the distance between the heel markers at heel contact of the leading foot and the trailing foot [26]. Step width (cm) was defined as the lateral distance between the midpoint of the toe and heel markers of the two feet during the double stance phase.[26] Step time (s) was defined as the time elapsed from the heel marker contact of successive footsteps [21]. Swing time (s) was defined as the time elapsed between the last contact of the toe marker of one foot and the initial contact of the heel marker of the same foot [21].

### Statistical analysis

SPSS software (version 21.0, IBM Corporation, Chicago, IL) was used to perform the statistical analyses. The Shapiro-Wilk test was conducted to test data normality. Descriptive statistics were used to report the demographic profiles of participants for the MCI and control groups. An independent sample t-test was used to compare the demographic profile between the two groups. A two groups (MCI, Control) x 2 walking conditions (SF, FS) mixed model repeated measures ANOVA was conducted to assess significant main effects and interactions. LSD was used for the post-hoc analysis and a probability level of 0.05 was set to denote statistical significance.

## Results

### Participant characteristics

The demographic, anthropometric, mobility, health, and cognitive characteristics of the participants in the two groups are summarized in Table 1. There were no significant differences between groups for the demographic, mobility, and health measures, except participants with MCI took longer to complete the TUG test than the controls (Table 1). The mean values for the self-selected slow, usual, and fast baseline walking speeds were similar between the two groups (p > 0.05). As expected, the older adults in the MCI group had lower scores on the global cognitive function test (MSET10, MoCA) (p = 0.001) and the tests of attention (DST, TMT A) and executive function (TMT B-A) than those in the control group (p = 0.001).

**Table 1:** Demographic, anthropometric, mobility, health, and cognitive characteristics of the participants (N = 50).

Characteristics	Control group (n = 25)	MCI group (n = 25)	p-value
Age (yrs)	68.72 ± 4.67	68.56 ± 3.79	0.89
Gender (Male: Female)	4:21	4:21	1.00
BMI (kg/m^2^)	22.38 ± 3.09	23.40 ± 2.77	0.23
Number of medications	0.92 ± 1.00	1.12 ± 1.17	0.52
At least 1 fall in the past year (n)	8	7	0.84
Leg length (cm)			
*Right side*	79.72 ± 5.00	80.12 ± 4.76	0.77
*Left side*	79.71 ± 4.94	80.14 ± 4.82	0.76
TUG (s)	7.02 ± 0.88	7.84 ± 1.23	0.01^a^
Gait speed (m/s)			
*Slow gait speed*	0.95 ± 0.16	0.99 ± 0.21	0.52
*Usual gait speed*	1.12 ± 0.15	1.07 ± 0.12	0.17
*Fast gait speed*	1.45 ± 0.14	1.39 ± 0.21	0.27
FES-I (score, total score = 64)	26.12 ± 8.90	28.60 ± 9.43	0.34
GDS (score, total score = 15)	1.40 ± 1.44	1.48 ± 1.50	0.85
MSET10 (score, total score = 29)	27.64 ± 1.35	25.08 ± 2.10	0.001^a^
MoCA (score, total score = 30)	26.04 ± 2.01	19.56 ± 2.26	0.001^a^
DST (score, total score = 28)	15.28 ± 3.63	11.56 ± 2.65	0.001^a^
TMT A (s)	45.83 ± 21.80	64.88 ± 19.11	0.001^a^
TMT B-A (s)	40.30 ± 21.90	164.62 ± 158.25	0.001^a^

Data are presented as means and SD except for gender and fall history. Independent sample t-test for continuous data and Chi-square test for categorical data.

^a^Significant difference at p < 0.05.

TUG = Timed Up and Go test; FES-I = Falls Efficacy Scale-International; MSET10 = Mental State Examination T10; MoCA = Montreal Cognitive Assessment; GDS-15 = Geriatric Depression Scale-15; DST = Digit Span Test; TMT A = Trail Making Test Part A; TMT B-A = subtracting Part B from Part A.

### Gait speed before and after the transition phases

For the SF condition, gait speed at the initial slow pace (MCI group = 0.86 ± 0.18 m/s, Control group = 0.83 ± 0.13 m/s, p = 0.52) and after reaching the fast pace (MCI group = 1.66 ± 0.26 m/s, Control group = 1.67 ± 0.24 m/s, p = 0.91) were not significantly different between the MCI and control groups. Similarly, for the FS condition, gait speed at the initial fast pace (MCI group = 1.65 ± 0.25 m/s, Control group = 1.70 ± 0.23 m/s, p = 0.57) and after reaching the slow pace (MCI group = 0.84 ± 0.19 m/s, Control group = 0.87 ± 0.13 m/s, p = 0.46) did not differ significantly between the two groups. There was also no significant difference for total distance walked during the slow and fast speed transitions between the groups (p = 0.07).

### Spatiotemporal gait parameters during gait speed transitions

Mean and variability values for the spatiotemporal parameters are shown in Table 2. Mixed-model repeated-measures ANOVA revealed no significant interactions or group effects but significant condition effects for step length, step width, and step time. Specifically, participants in both groups exhibited shorter step lengths and step times and greater step widths in the FS condition compared with the SF condition (p < 0.05). Main and interaction effects were not found for swing time.

**Table 2:** Means and variability of spatiotemporal parameters of the MCI and control groups during SF and FS conditions.

Parameters	MCI group (n = 25)		Control group (n = 25)		p-value		
	SF	FS	SF	FS	Group	Condition	Interaction
**Means spatiotemporal parameters**							
Step length (cm)	62.18 ± 9.18	59.57 ± 8.44	63.05 ± 6.91	61.77 ± 7.13	0.48	0.01^a^	0.32
Step width (cm)	7.06 ± 1.93	7.56 ± 2.19	7.51 ± 2.50	7.96 ± 2.76	0.52	0.01^a^	0.89
Step time (s)	0.51 ± 0.04	0.48 ± 0.04	0.51 ± 0.03	0.50 ± 0.04	0.21	0.001^a^	0.16
Swing time (s)	0.46 ± 0.03	0.46 ± 0.04	0.46 ± 0.04	0.46 ± 0.04	0.89	0.84	0.50
**Variability of spatiotemporal parameters, Coefficient of variation**							
Step length variability	10.00 ± 3.97	13.93 ± 5.38	10.65 ± 3.02	11.12 ± 3.15	0.24	0.002^a^	0.01^a^
Step width variability	37.17 ± 10.43	37.10 ± 10.89	34.10 ± 10.38	33.98 ± 10.78	0.20	0.96	0.99
Step time variability	12.75 ± 3.67	9.63 ± 3.57	13.78 ± 3.07	10.71 ± 3.29	0.21	0.001^a^	0.97
Swing time variability	13.32 ± 6.01	13.35 ± 6.01	13.53 ± 4.52	10.43 ± 2.87	0.24	0.08	0.03^a^

Data are expressed as means and SD. Group × Walking condition interaction as calculated by using a 2 groups x 2 walking conditions mixed model repeated measures ANOVA.

^a^Significant difference at p < 0.05.

SF = Slow to fast speed condition; FS = Fast to slow speed condition.

The mixed-model repeated-measure ANOVAs yielded group x walking condition interactions for step length (p = 0.01) and swing time (p = 0.03) variability with the post-hoc comparisons revealing these spatiotemporal variability measures were significantly higher in the MCI group compared with the control group in the FS condition (p = 0.03). A significant condition effect indicated step time variability for all participants was significantly higher in the SF condition compared with the FS condition (p = 0.001). There were no condition or interaction effects for step width variability.

## Discussion

The aim of this study was to contrast gait characteristics during slow and fast speed transitions between older adults with MCI and cognitively intact controls. In line with our hypotheses, we found gait variability was significantly higher in the MCI group than the control group in the fast to slow speed walking condition, and that mean spatiotemporal parameters did not differ between the groups in both fast to slow and slow to fast gait speed transitions [10,27]. However, contrary to our hypotheses, we did not find gait variability to be significantly higher in the MCI group than the control group in the slow to fast speed walking condition. Walking is an everyday activity that places requires cognitive and motor resources to stop and start, turn, adjust speed, and negotiate hazards [5]. For example, compared to normal steady walking, abruptly decelerating gait speed involves greater neuromuscular and cognitive integration to quickly arrest the body’s forward momentum, and it is likely that this ability to abruptly slow down is crucial for avoiding falls in everyday life. Step length variability is considered to be a measure of spatial domain integrity and rhythmic stepping, [23] and high step length variability is associated with lower grey matter integrity in areas responsible for memory and executive function (the hippocampus and anterior cingulate gyrus) in older adults [28]. High swing time variability is also considered to be an indicator of gait dysrhythmicity and instability and a robust predictor of falls [24,29]. Therefore, the greater gait variability observed in the older adults with MCI compared to controls found during FS transitions may reflect inefficient gait regulation of the body momentum during rapid deceleration, and compliments a previous finding that older adults with MCI have impaired ability to terminate gait and are at increased risk of falls [11].

Both the older adults with MCI and their healthy counterparts had greater step time variability under slow to fast transitions compared to fast to slow transitions. This finding is consistent with a previous study that reported an increase in step time variability in older adults with and without MCI when rapidly accelerating the body from a static to dynamic state (gait initiation), [10] and likely reflects the difficulty of controlling gait when abruptly propelling the body forward. However, the finding that all measures of gait variability did not differ between the older adults with MCI and the healthy controls during the slow to fast speed condition was contrary to our hypotheses and may indicate this transition is a more practiced task in everyday life and thus less able to reveal gait deficits in participants with MCI.

As expected, the MCI group had poorer attention and executive function than the control group; early disabling symptoms of MCI [30]. Previous studies have found poor attention and executive function are associated with impaired gait in complex gait tasks such as dual-task walking, walking a curved path and undertaking a turning to walk transition task in older adults [31–34]. These cognitive deficits may also explain, at least in part, why older adults with MCI have increased gait variation in fast to slow gait speed transitions.

The finding that older adults with MCI have trouble regulating gait pattern during an abrupt fast to slow speed transition suggests that their risk of falls may be increased while encountering real-life discrete changes in environmental conditions. Thus, the fast to slow speed transition task should be integrated as part of comprehensive fall risk assessment in individuals with MCI. Moreover, the present findings convey helpful strategic intervention for people with MCI by adding gait speed transition training particularly from fast to slow to minimize the risk of falling.

Some limitations in this study need to be acknowledged. We measured gait performance under controlled laboratory conditions rather than in a real-life walking situation. Therefore, complementary assessments of gait speed transitions with environmental distractions and/or while participants perform a secondary cognitive or physical task would provide a greater challenge to gait control and possibly amplify differences between older people with and without MCI. Further, our study outcomes were restricted to parameters. Future studies could include kinematic and kinetic data to provide further insight into postural control of older adults with MCI during gait speed transitions.

## Conclusions

The present study demonstrated that older adults with MCI had greater step length and swing time variability when undertaking abrupt fast to slow speed transitions as compared to cognitively intact controls. This assessment could be integrated into fall risk assessments to elucidate gait impairment and fall risk in older adults with MCI.

## Supporting information

S1 File Spatiotemporal parameters during gait speed transitions of participants in the MCI and control groups. Variability and means of spatiotemporal parameters for each individual during slow and fast speed transitions and gait speed before and after gait speed transitions.
(PDF)
