# Supplementary material for: Gait variability during abrupt slow and fast speed transitions in older adults with mild cognitive impairment
Source: PLoS One. 2022 Oct 21;17(10):e0276658. doi: 10.1371/journal.pone.0276658 (PMC9586342; doi:10.1371/journal.pone.0276658)
Supplement: S1 File — Variability and means of spatiotemporal parameters for each individual during slow and fast speed transitions and gait speed before and after gait speed transitions. (PDF) [file pone.0276658.s001.pdf]

ID	Group	Variability of Spatiotemporal Parameters							
		Slow to Fast Speed Condition				Fast to Slow Speed Condition			
		Step length	Step width	Step time	Swing time	Step length	Step width	Step time	Swing time
Control01	1	8.592	47.393	15.031	15.595	12.595	35.721	12.074	10.357
Control02	1	10.444	28.947	12.494	20.372	10.967	22.944	4.831	12.340
Control03	1	14.503	29.640	19.406	13.226	11.476	35.170	17.888	10.143
Control04	1	9.063	22.381	14.607	16.046	15.767	45.004	11.477	11.925
Control05	1	10.369	22.111	10.803	17.229	8.442	31.225	10.651	7.163
Control06	1	11.705	31.209	13.097	14.966	12.426	50.206	12.513	11.766
Control07	1	10.974	47.431	10.566	10.458	10.460	54.057	8.809	15.114
Control08	1	10.041	48.770	10.473	9.617	10.828	48.575	13.660	9.639
Control09	1	10.205	41.982	16.986	22.978	7.283	32.794	12.407	12.200
Control10	1	8.723	27.788	10.290	9.739	8.390	35.721	9.753	10.225
Control11	1	7.052	23.290	13.453	9.115	6.579	20.859	7.024	7.430
Control12	1	15.509	21.293	17.838	11.762	13.953	28.652	9.482	9.098
Control13	1	17.192	52.983	16.735	12.510	15.272	46.559	10.928	10.647
Control14	1	7.137	41.835	15.347	15.783	16.478	36.432	7.837	9.372
Control15	1	13.349	28.599	13.605	18.723	12.611	23.461	10.443	17.445
Control16	1	4.250	32.535	8.981	7.724	6.250	35.721	5.470	5.014
Control17	1	7.232	37.771	12.029	10.541	10.577	26.429	10.820	9.964
Control18	1	10.166	57.219	13.571	8.743	10.041	20.102	17.689	12.218
Control19	1	10.837	33.302	9.147	7.569	15.196	43.730	7.994	7.529
Control20	1	8.540	32.449	11.366	7.574	9.281	29.191	8.664	5.214
Control21	1	9.144	29.577	16.586	20.375	13.100	19.200	12.749	12.340
Control22	1	11.788	19.196	20.755	10.057	6.837	14.634	14.853	12.340
Control23	1	10.096	32.956	13.346	17.521	7.100	28.522	8.120	11.793
Control24	1	14.390	34.962	13.964	17.892	15.717	46.970	13.221	12.340
Control25	1	14.892	27.005	13.954	12.151	10.265	37.546	8.284	7.183
Means	1	10.65	34.10	13.78	13.53	11.12	33.98	10.71	10.43
SD	1	3.02	10.38	3.07	4.52	3.15	10.78	3.29	2.87
Max	1	17.19	57.22	20.76	22.98	16.48	54.06	17.89	17.45
Min	1	4.25	19.20	8.98	7.57	6.25	14.63	4.83	5.01
MCI01	2	12.813	37.598	12.309	11.117	15.262	33.122	11.022	7.744
MCI02	2	7.034	33.867	8.404	6.252	6.305	11.773	14.073	23.683
MCI03	2	9.259	36.589	17.874	18.739	9.323	39.815	14.526	11.805
MCI04	2	4.986	31.855	11.931	10.045	10.859	45.470	8.901	14.280
MCI05	2	4.276	49.598	14.389	17.620	15.907	41.282	7.656	7.315
MCI06	2	5.804	27.134	11.520	11.752	8.679	49.925	5.492	5.603
MCI07	2	14.433	28.573	10.344	7.562	16.744	34.185	7.009	4.925
MCI08	2	11.036	35.316	11.154	9.145	21.952	30.946	9.913	13.810
MCI09	2	11.698	45.215	8.248	13.470	21.201	38.867	10.482	19.608
MCI10	2	21.639	36.234	16.119	13.941	15.045	48.219	10.545	20.789
MCI11	2	17.115	25.220	12.990	14.620	25.055	34.942	13.410	9.632
MCI12	2	9.687	43.521	9.835	7.754	11.351	24.132	7.318	17.071
MCI13	2	10.013	48.672	6.490	27.464	15.396	33.316	5.775	26.338
MCI14	2	5.096	24.627	14.049	8.630	18.380	26.759	11.961	6.790
MCI15	2	7.174	43.754	13.078	17.379	11.922	38.867	6.377	12.779
MCI16	2	8.618	15.867	11.425	11.286	15.122	33.874	4.961	6.745
MCI17	2	8.164	52.553	10.375	9.301	8.608	31.361	6.275	6.480
MCI18	2	9.747	59.746	14.232	10.170	12.074	61.532	14.523	11.276
MCI19	2	10.433	31.881	11.288	8.928	22.491	38.073	5.654	18.932
MCI20	2	13.345	29.016	12.217	18.811	12.562	47.608	18.011	10.052
MCI21	2	9.050	28.848	16.920	19.726	5.691	26.973	10.910	14.432
MCI22	2	8.075	32.147	17.044	19.994	17.879	58.879	7.426	22.108
MCI23	2	13.818	52.952	23.367	26.318	4.875	38.867	14.080	11.546
MCI24	2	7.412	41.246	14.799	6.421	10.168	27.184	7.138	15.473
MCI25	2	9.284	37.346	8.359	6.430	15.464	31.542	7.410	14.640
Means	2	10.000	37.175	12.750	13.315	13.933	37.101	9.634	13.354
SD	2	3.967	10.426	3.673	6.011	5.382	10.893	3.575	6.013
Max	2	21.639	59.746	23.367	27.464	25.055	61.532	18.011	26.338
Min	2	4.276	15.867	6.490	6.252	4.875	11.773	4.961	4.925

ID	Means of Spatiotemporal Parameters							
	Slow to Fast Speed Condition				Fast to Slow Speed Condition			
	Step length (cm)	Step width (cm)	Step time (s)	Swing time (s)	Step length (cm)	Step width (cm)	Step time (s)	Swing time (s)
Control01	68.225	4.787	0.491	0.383	64.488	4.323	0.473	0.373
Control02	54.960	7.386	0.469	0.378	58.089	7.553	0.426	0.446
Control03	77.692	11.635	0.501	0.410	79.626	11.241	0.482	0.404
Control04	66.619	10.383	0.459	0.410	68.387	12.232	0.474	0.431
Control05	62.176	8.813	0.504	0.450	62.869	7.711	0.459	0.438
Control06	55.144	6.707	0.567	0.447	59.219	5.613	0.545	0.470
Control07	54.864	3.391	0.549	0.488	53.253	4.178	0.542	0.488
Control08	56.422	5.761	0.529	0.472	55.409	5.948	0.541	0.504
Control09	66.641	7.104	0.531	0.464	66.452	7.265	0.533	0.479
Control10	73.432	6.177	0.563	0.512	68.897	6.378	0.581	0.539
Control11	65.068	11.652	0.496	0.422	59.347	14.012	0.506	0.442
Control12	56.353	10.956	0.548	0.454	52.205	11.072	0.520	0.454
Control13	60.940	6.672	0.486	0.481	60.912	6.668	0.464	0.461
Control14	58.566	5.470	0.472	0.407	55.035	6.486	0.476	0.417
Control15	66.074	10.072	0.519	0.557	59.650	11.406	0.538	0.483
Control16	46.216	4.232	0.492	0.459	44.947	4.837	0.482	0.438
Control17	63.692	6.663	0.520	0.481	60.222	6.192	0.524	0.489
Control18	64.537	4.299	0.538	0.484	63.081	5.280	0.539	0.491
Control19	69.527	5.571	0.496	0.438	64.767	7.249	0.469	0.425
Control20	69.314	7.398	0.566	0.481	64.003	7.684	0.551	0.492
Control21	61.422	7.741	0.511	0.462	59.654	10.531	0.502	0.483
Control22	57.636	12.265	0.519	0.465	60.768	10.832	0.467	0.479
Control23	67.462	8.555	0.524	0.488	68.712	11.041	0.492	0.476
Control24	65.081	5.923	0.518	0.531	60.599	5.328	0.544	0.483
Control25	68.103	8.130	0.478	0.407	73.610	7.974	0.447	0.395
Means	63.05	7.51	0.51	0.46	61.77	7.96	0.50	0.46
SD	6.91	2.50	0.03	0.04	7.13	2.76	0.04	0.04
Max	77.69	12.26	0.57	0.56	79.63	14.01	0.58	0.54
Min	46.22	3.39	0.46	0.38	44.95	4.18	0.43	0.37
MCI01	70.608	9.239	0.541	0.477	66.556	11.134	0.520	0.460
MCI02	64.889	10.848	0.506	0.445	65.653	13.062	0.463	0.464
MCI03	80.161	7.819	0.526	0.482	75.698	6.535	0.541	0.510
MCI04	72.402	5.766	0.458	0.395	69.094	8.071	0.432	0.365
MCI05	69.691	4.251	0.529	0.466	64.822	5.646	0.500	0.448
MCI06	56.602	5.621	0.445	0.414	56.177	6.012	0.467	0.414
MCI07	49.050	9.624	0.460	0.427	48.095	9.015	0.444	0.404
MCI08	49.746	7.327	0.489	0.436	50.443	7.064	0.452	0.426
MCI09	52.070	4.919	0.468	0.430	52.753	6.401	0.476	0.510
MCI10	51.623	7.392	0.506	0.512	41.374	8.876	0.451	0.478
MCI11	63.985	9.254	0.478	0.458	52.491	9.318	0.483	0.455
MCI12	61.965	9.386	0.506	0.470	57.605	10.776	0.468	0.481
MCI13	53.204	7.116	0.503	0.468	55.518	8.326	0.462	0.465
MCI14	72.452	7.425	0.587	0.508	52.382	8.572	0.490	0.494
MCI15	62.118	4.155	0.515	0.510	58.834	4.204	0.498	0.494
MCI16	47.372	9.716	0.551	0.510	48.433	8.303	0.476	0.465
MCI17	58.502	5.008	0.527	0.477	55.438	7.323	0.531	0.456
MCI18	70.379	6.174	0.508	0.471	70.295	6.145	0.520	0.512
MCI19	69.876	5.285	0.437	0.447	68.621	6.243	0.416	0.466
MCI20	69.873	5.810	0.611	0.495	67.651	5.766	0.604	0.523
MCI21	57.072	9.420	0.456	0.409	60.841	8.508	0.484	0.430
MCI22	57.211	6.710	0.555	0.486	56.072	4.051	0.490	0.410
MCI23	52.945	4.788	0.518	0.456	63.538	4.402	0.440	0.427
MCI24	72.702	6.626	0.495	0.461	68.172	6.686	0.524	0.472
MCI25	67.939	6.937	0.491	0.424	62.784	8.679	0.477	0.416
Means	62.177	7.065	0.507	0.461	59.574	7.565	0.484	0.458
SD	9.179	1.929	0.042	0.033	8.436	2.188	0.040	0.039
Max	80.161	10.848	0.611	0.512	75.698	13.062	0.604	0.523
Min	47.372	4.155	0.437	0.395	41.374	4.051	0.416	0.365

ID	Gait Speed Before and After the Transition Phases			
	Slow to Fast Speed Condition		Fast to Slow Speed Condition	
	Initial Slow Pace (m/s)	Reaching Fast Speed (m/s)	Initial Fast Pace (m/s)	Reaching Slow Speed (m/s)
Control01	0.847	1.782	1.862	1.039
Control02	0.805	1.685	1.752	1.039
Control03	0.783	2.354	2.344	1.063
Control04	1.087	1.956	1.968	1.004
Control05	0.807	1.631	1.717	0.990
Control06	0.606	1.467	1.663	0.713
Control07	0.667	1.372	1.409	0.753
Control08	0.754	1.420	1.370	0.720
Control09	0.915	1.573	1.642	0.876
Control10	0.880	1.710	1.726	0.767
Control11	0.844	1.703	1.631	0.854
Control12	0.680	1.295	1.319	0.684
Control13	0.793	1.737	1.819	0.901
Control14	0.915	1.669	1.634	0.871
Control15	0.674	1.763	1.701	0.729
Control16	0.743	1.205	1.247	0.750
Control17	0.919	1.652	1.638	0.802
Control18	0.686	1.656	1.731	0.724
Control19	1.031	1.881	1.913	0.951
Control20	0.849	1.704	1.686	0.828
Control21	0.941	1.517	1.703	0.875
Control22	0.716	1.487	1.608	0.831
Control23	0.845	1.800	1.786	1.056
Control24	0.757	1.827	1.663	0.735
Control25	1.120	1.939	1.989	1.139
Means	0.827	1.671	1.701	0.868
SD	0.131	0.238	0.227	0.134
Max	1.120	2.354	2.344	1.139
Min	0.606	1.205	1.247	0.684
MCI01	0.934	1.966	1.914	0.962
MCI02	0.882	1.796	1.676	0.969
MCI03	0.824	2.158	2.096	0.896
MCI04	1.323	2.209	2.082	1.120
MCI05	0.952	1.677	1.720	0.908
MCI06	0.940	1.549	1.575	1.018
MCI07	0.740	1.538	1.619	0.814
MCI08	0.660	1.300	1.439	0.743
MCI09	0.872	1.445	1.424	0.783
MCI10	0.756	1.423	1.374	0.564
MCI11	0.665	1.926	1.609	0.678
MCI12	0.811	1.558	1.580	0.878
MCI13	0.907	1.464	1.501	0.789
MCI14	0.593	1.542	1.529	0.542
MCI15	0.895	1.614	1.586	0.820
MCI16	0.614	1.131	1.230	0.338
MCI17	0.791	1.351	1.377	0.763
MCI18	1.084	2.051	2.058	0.887
MCI19	1.190	1.919	2.121	1.164
MCI20	0.723	1.615	1.659	0.800
MCI21	0.744	1.656	1.554	0.998
MCI22	0.791	1.536	1.543	0.613
MCI23	0.679	1.732	1.817	0.994
MCI24	0.981	1.718	1.779	0.944
MCI25	1.028	1.706	1.404	1.044
Means	0.855	1.663	1.651	0.841
SD	0.177	0.265	0.246	0.191
Max	1.323	2.209	2.121	1.164
Min	0.593	1.131	1.230	0.338
